# Unconventional Increase in Non-Radiative Transitions in Plasmon-Enhanced Luminescence: A Distance-Dependent Coupling

**DOI:** 10.1038/srep36691

**Published:** 2016-11-16

**Authors:** Eder José Guidelli, Ana Paula Ramos, Oswaldo Baffa

**Affiliations:** 1 Departamento de Física, Faculdade de Filosofia, Ciências e Letras de Ribeirão Preto, Universidade de São Paulo, Av. Bandeirantes, 3900, 14040-901, Ribeirão Preto, SP, Brasil; 2 Departamento de Química, Faculdade de Filosofia, Ciências e Letras de Ribeirão Preto, Universidade de São Paulo, Av. Bandeirantes, 3900, 14040-901, Ribeirão Preto, SP, Brasil

## Abstract

We used Optically Stimulated Luminescence (OSL) from X-ray-irradiated sodium chloride nanocrystals to investigate how silver nanoparticle (AgNP) films enhanced luminescence. We controlled the emitter-AgNP distance and used the OSL intensity and decay times to explore the plasmonic interactions underlying the enhanced luminescence. Both intensity and decay times depended on the emitter-AgNP distance, which suggested that a mechanism involving energy transfer from the localized surface plasmons (LSPs) to the trapped electrons took place through a distance-dependent coupling. Compared to other plasmon-enhanced mechanisms, the energy transfer observed here occurred in the opposite bias: LSP relaxation stimulated electron transfer from non-optically active traps to optically active traps, which culminated in enhanced emission. Therefore, a different mechanism of plasmonic coupling converted optically unreachable electrons into useful luminescence information.

Localized surface plasmons (LSPs) are electronic oscillations at nanostructured metal surfaces caused by an incident light beam of appropriate resonant wavelengths. Interaction of LSPs with the surrounding medium generates new optical properties that enhance several luminescent processes^1^,^2^,^3^,^4^,^5^,^6^, giving rise to the so-called plasmon-enhanced luminescence.

Such luminescence enhancement usually originates via two mechanisms: (1) the excitation mechanism, which results from amplification of the local electric field close to the metal surface; and (2) the metal-coupled emission mechanism, which involves energy transfer from the excited luminophore to the plasmons, to increase the radiative decay rates and reduce the lifetime of the plasmophore emission^7^,^8^,^9^. The excitation mechanism usually occurs at very short distances (<10 nm) from the metal surface, whereas the metal-coupled emission mechanism involves a distance-dependent relationship between the plasmon and the luminophore system^7^,^8^. However, the mechanisms underlying enhanced luminescence remain a matter of debate^8^. In this article, we used X-ray-irradiated sodium chloride (NaCl) nanocrystals and Optically Stimulated Luminescence (OSL) to investigate how silver nanoparticle (AgNP) films enhanced luminescence. We will show that a third mechanism consisting of a distance-dependent coupling may also occur during the excitation process. In this mechanism, LSP relaxation excites electrons and stimulates their transfer from non-optically active traps to optically active traps, leading to enhanced OSL.

The samples analyzed in this study consisted of NaCl nanocrystals deposited over AgNP films and irradiated with a 10-Gy X-ray dose. X-rays create defects in the NaCl nanocrystals. These defects can be subsequently read out upon stimulation with a blue LED light (470 nm), and the stimulated emission (OSL) is collected in the detection bandwidth 270–370 nm^10^,^11^,^12^. Both the OSL stimulation and the readout bandwidth overlap with the silver plasmon band, thereby favoring plasmonic coupling.

Figure 1 illustrates the normalized (inset) and non-normalized OSL signals from NaCl crystals deposited on glass and over the AgNP films containing 3, 5, and 10 AgNP/chitosan bilayers. A detailed description of the procedures used to prepare and grow the films and of the optical and morphological characterization of the films can be found elsewhere^13^. According to Fig. 1, the AgNP films enhanced the OSL emission, and the enhancement factor was proportional to the number of AgNP layers. AgNP films deposited on aluminum substrates led to considerably smaller OSL enhancements^13^, which suggested that possible defects created in the NaCl/AgNP film interface did not enhance the OSL emission.

Figure 2(a) shows that the OSL intensity (integral of the curve) increased linearly as a function of the optical density of the films at 470 nm (the wavelength of maximum OSL stimulation) increased linearly as a function of the optical density of the films. This linear behavior agreed with the hypothesis that increased local electric fields in the vicinity of the AgNP films under plasmon resonance conditions enhanced the OSL emission^14^. In this sense, the more intense the plasmon resonance band, the larger the local electric field and the more enhanced the OSL emission. Figure 2(b) depicts the initial OSL intensity as a function of the optical density of the films. Compared to the OSL enhancement in terms of the integral of the OSL curve, the AgNP films caused very low, non-significant enhancement of the initial OSL intensity.

Local intensification of the electric field close to the metal nanoparticle surface should increase the rate at which trapped electrons are released (excitation) and consequently accelerate the OSL decay. However, Fig. 1 evidenced that a larger number of AgNP/chitosan bilayers slowed the OSL curve decay. Therefore, the increased excitation rate resulting from a more intense electric field close to the AgNP films (mechanism 1) may not have been the only cause of OSL enhancement.

To investigate the interaction of the NaCl nanocrystals with the AgNP films even further, 0.5, 1, 2, 4, and 6 chitosan/poly(acrylic acid) bilayers were deposited between the AgNP film and the NaCl nanocrystals, which increased the distance between the metal film and the emitter nanocrystals. The thickness of one chitosan/poly(acrylic acid) bilayer was estimated as being 15 ± 2 nm on the basis of atomic force microscopy image (not shown here). Assuming that the polymeric film grew linearly, the spacing between the NaCl nanocrystals and the AgNP film varied from 0 to 90 nm (0 to 6 bilayers).

Figure 3(a) brings the OSL intensity (integral) as a function of the distance between the NaCl nanocrystals and the AgNP film, relative to the NaCl nanocrystals deposited over glass. This experiment was repeated five times, including the growth of the AgNP film and of the chitosan/poly(acrylic acid) bilayers, sample irradiation, and OSL measurements (at least three different samples for each interlayer spacing, for each repetition). The error bars represent the standard deviation obtained from the five repetitions. The maximum OSL intensity occurred when the AgNP films and the NaCl nanocrystals were 15 nm away from each other (one bilayer). The OSL intensity for a distance of 30 nm was still slightly higher than the intensities obtained for a distance of ≈7.5 nm (one chitosan layer) and the intensities recorded when the NaCl nanocrystals were deposited directly over the AgNP films. For distances larger than 30 nm (two bilayers), the OSL intensity decayed to a minimum value for the largest tested distance (90 nm–six chitosan/poly(acrylic acid) bilayers), but this minimum value was still twofold larger as compared to the OSL intensity obtained when the NaCl nanocrystals were deposited over glass.

Figure 3(b) revealed that the OSL curve started to decay faster with increasing distance between the NaCl nanocrystals and the AgNP film. In other words, the slow OSL decay pointed out in Fig. 1 only occurred when the luminescent nanocrystals were deposited at short distances from the metal film surface. The inset in Fig. 3(b) shows that the OSL decay times (τ_1_, τ_2_, and τ_3_ normalized by the respective decay times of NaCl nanocrystals deposited over glass) decreased as a function of the distance from the AgNP films.

Figure 4(a) presents the OSL signal recorded for the NaCl nanocrystals deposited over glass during a first complete OSL readout (black line) and during a second measurement (red line) carried out 10 minutes after the first readout. At the end of the first measurement (30 s of OSL stimulation), the OSL intensity vanished completely. However, the initial intensity of the second readout was around 8% of the initial intensity of the first measurement; in other words, the OSL intensity was partially regenerated, a known characteristic of NaCl crystals^15^. The inset in Fig. 4(a) provided further evidence that the OSL signal regenerated. In contrast, Fig. 4(b) did not reveal any regeneration effect for the NaCl nanocrystals deposited over the AgNP films, and the second measurement only showed background noise (red line). This result suggested that all the trapped electrons were released during the first OSL measurement under plasmon resonance conditions.

On the basis of Fig. 2, the OSL intensified as a function of the plasmon band intensity; hence, a more intense electric field may have made the release of trapped electrons more efficient under plasmon resonance conditions. Nevertheless, more efficient electron release should have resulted in faster OSL intensity decay, (i.e. smaller decay times should be observed), which was not the case here. Increasing the number of AgNP/Chitosan bilayers elicited a more intense plasmon resonance band (shown in Fig. 2(a) as the optical density at 470 nm). Therefore, Fig. 1 suggested that a more intense plasmon resonance band slowed the OSL decays. In this sense, intensification of the electric field at the film surface might not have been the only mechanism involved in OSL enhancement. Furthermore, the enhanced luminescence caused by intensification of the electric field around the metal nanoparticles should occur for distances of up to 10 nm from the nanoparticle surface^7^,^8^. Because (i) the maximum OSL intensity was detected for a distance of 15 nm and (ii) a twofold enhancement was still observed for an interlayer spacing around 90 nm (six chitosan/poly(acrylic acid) bilayers), as shown in Fig. 3(a), intensification of the local electric field may not have been the single cause of OSL enhancement, and a second mechanism may have taken place.

Electrodynamic theory has shown that energy transfer to plasmons can happen for distances of up to 400 nm between the luminescent center/dipole and the metal film^7^,^16^, with maximum radiative decay rates being obtained around 60 nm^16^. This metal-coupled emission occurs via an energy transfer mechanism similar to the mechanism described by Förster^7^—in this mechanism, energy is transferred from the excited luminescent center (donor) to the acceptor (LSPs in this case) followed by far-field emission. Hence, in the specific case of optically stimulated luminescence, OSL stimulation with a wavelength that matched the AgNP plasmon band (470 nm in this case) intensified the local field and enhanced the release of trapped electrons; i.e., the excitation rate increased (mechanism 1 as described in the introduction). Thereafter, the released electrons recombined with trapped holes. According to the OSL model, electron-hole recombination leads to a luminescent center in an excited state^12^. In the absence of the AgNP films, the excited luminescent center relaxes to the ground state, giving rise to the OSL emission. Assuming that an energy transfer mechanism occurred in our OSL system, the electron-hole recombination characteristic of the OSL process prompted the excited luminescent center to transfer its energy to the localized surface plasmons, which then irradiated the energy to the far field. Although this energy-transfer mechanism (mechanism 2 – as described in the introduction) may have taken place in our NaCl/AgNP multilayered system, it could not account for the increased OSL decay times pointed out in Figs 1 and 3(b). Indeed, according to the literature, this energy transfer should decrease the luminescence lifetime, which in turn should reduce (or at least not increase) the OSL decay times^17^. Together, these observations suggested that a third mechanism might have caused OSL enhancement.

As depicted in Fig. 4, irradiated NaCl nanocrystals display an unconventional physical property: the OSL signal regenerates some minutes after one complete OSL readout^15^. BaFBr:Eu^2+^ also presents this unusual luminescence recovery^18^. It is possible to rationalize this phenomenon by considering the existence of (i) optically active traps that can release trapped electrons during the OSL readout, and (ii) non-optically active traps that are inactive during the OSL stimulation, but which can act as reservoirs of charges that fill emptied optically active traps^15^. In other words, upon release of electrons from optically active traps, electrons trapped in non-optically active traps can be transferred to the optically active traps. This non-radiative electron transfer partially regenerates the OSL signal^15^.

As for the NaCl nanocrystals deposited over glass, the OSL curve decayed faster due to rapid emptying of the optically active traps. Charge transfer from non-optically active to optically active traps is very slow and takes up to a few minutes^15^. For this reason, the maximum regenerated OSL signal was observed around 10 minutes after the first OSL measurement^15^. For the NaCl nanocrystals deposited over the AgNP films, the enhanced OSL signal in the first measurement and the absence of regenerated OSL signal in the second measurement (10 minutes later) revealed that all the electrons were released during the first OSL stimulation. In this case, even electrons trapped in non-optically active traps were released and transferred to recently emptied optically active traps, thereby suggesting that this non-radiative electronic transition enhanced under plasmon resonance conditions.

Concerning the NaCl deposited over the AgNP films, electron transfer from non-optically active to optically active traps during the OSL stimulation increased the OSL decay time—the optically active traps kept being filled until non-optically active traps were completely emptied. This meant that longer times were necessary to empty the optically active traps. According to Fig. 3(b), the OSL curve started to decay faster again upon increasing distance between the NaCl nanocrytals and the AgNP film. This provided further evidence that distance-dependent plasmonic interactions close to the AgNP surface enhanced the non-radiative transition of electrons from non-optically active to optically active traps. Compared to the decay times of the NaCl nanocrystals over glass, the normalized values of τ_1_, τ_2_, and τ_3_ at different distances of the NaCl nanocrystals from the AgNP films (as depicted in the inset of Fig. 3(b)) were similar to the enhancement of the OSL intensity observed in Fig. 3(a). This suggested that faster electron transition from non-optically active to optically active traps enhanced the OSL.

Taken together, the results of the present article suggest that electrons trapped in non-active traps could couple with localized surface plasmons through a non-radiative resonant energy transfer process. The localized surface plasmon dipole should excite electrons and stimulate their transfer from non-optically active to optically active traps. This electronic stimulation could also promote electron transfer from non-optically active traps directly to the conduction band or to any other trap and recombination center. However, in this case the de-trapping mechanism should be faster, and the OSL curve should reveal a much more significantly enhanced initial OSL intensity and a faster decay^17^, which was not the case here. This may also suggest that the plasmon-mediated non-radiative transition of electrons from non-optically active to optically active traps occurs because the AgNP plasmons have the amount of energy that is necessary to cause such electronic transition.

In summary, the enhanced OSL suggests that energy transfer from the LSPs to the trapped electrons through a distance-dependent coupling is also possible. This is the opposite bias of the energy transfer observed in systems where the metal-coupled emission mechanism takes place. This may represent a third mechanism and account for plasmon-enhanced luminescence processes in which optically unreachable electrons can be converted into useful luminescence information. Besides aiding the proposal of a different mechanism for better understanding of plasmon-enhanced luminescence processes, the results reported here reveal that LSPs are potentially applicable in systems and devices involving luminescence, charge storage, and transport, and that the OSL technique is a powerful tool to unravel plasmonic interactions. Further studies will focus on tuning the plasmon resonance band, which will allow for exploration of different electronic transitions.

## Materials and Methods

### LbL Multilayered Film Growth

The chemicals employed in the experiments were analytical reagent grade. All the chemicals were used as received. Silver nitrate (99.8%) was supplied by Cennabras; sodium borohydride was purchased from Sigma. All the aqueous solutions were prepared with purified Milli-Q^TM^ water. Silver nanoparticles (AgNPs) were produced by chemical reduction of silver nitrate^19^. To this end, AgNO_3_ aqueous solution (2 mmol L^−1^) was added to freshly prepared NaBH_4_ aqueous solution (4 mmol L^−1^). The system immediately became bright yellow, which indicated the formation of a colloidal dispersion. To ensure total reduction of the silver ions, the system was kept under vigorous stirring for 12 h. UV-Vis spectroscopy confirmed formation of AgNPs as seen from the plasmonic absorption peak at 390 nm. The average size of the AgNPs present in the freshly prepared colloidal dispersion was estimated as 30 nm by the dynamic light scattering technique (DLS). The zeta potential was −36 ± 2 mV.

Next, chitosan (0.1 wt.%) was dissolved in acetic acid solution (pH = 4). The solution was filtered through a Millipore^®^ membrane (0.45-μm pore size), and self-assembled films were produced by alternating the deposition of chitosan (positively charged) and AgNP (negatively charged) layers over glass (0.5 × 0.5 cm) by means of the layer-by-layer (LbL) technique^20^. The glass substrates were previously cleaned with a piranha solution and washed with Mili-Q^TM^ water and acetone. The first and the last layers consisted of the positively charged polyelectrolyte (chitosan). The substrates remained in each solution/dispersion for 20 min. All the layers were rinsed with Mili-Q^TM^ water for 5 min between each deposition step (to remove nonspecifically adsorbed chitosan and AgNPs) and dried under nitrogen flow. This procedure was repeated several times, to grow films with 3, 5, and 10 chitosan/AgNP bilayers.

Poly(acrylic acid) (0.2 wt.%) was dissolved at 60 °C in water. The chitosan/poly(acrylic acid) films were formed by deposition of alternating layers of chitosan and poly(acrylic acid) on the 3_AgNP films substrates by the LbL technique. Again, the substrates were immersed in each solution for 20 min, rinsed with Mili-Q^TM^ water for 5 min, and dried under nitrogen flow. This procedure was repeated several times, to grow films with 1, 2, 4, and 6 chitosan/poly(acrylic acid) bilayers.

After that, 10 μL of a NaCl solution (0.02 mol.L^−1^, water/acetone 20:80 (v/v)) was dropped over the deposited films and dried at 40 °C, to form the NaCl nanocrystals that would give rise to the X-Ray induced luminescence (OSL).

### Characterization

UV-Vis spectroscopy (transmission) was performed on a HP Vectra XM 5 Series 4 spectrophotometer. All the samples were irradiated with a dose of 10 Gy, in air. An X-Ray tube (Magnun - Moxtek, USA) operating at 48 kVp and 0.2 mA was employed. Optically stimulated luminescence (OSL) was acquired by using an OSL reader developed by the laboratory of Dosimetry and Nuclear Instrumentation of Universidade Federal de Pernambuco. Samples were excited with a blue LED with maximum emission at 470 nm. The detection system consisted of a photomultiplier and a Hoya U340 optical filter with transmittance in the 270–370 nm region. The OSL signals were recorded in triplicate (three different samples). No OSL signal emerged from the glass substrates (with or without deposited films) at the doses tested here. The error bars represent the experimental standard deviation for the three samples.

OSL has a physical principle similar to the principle of thermoluminescence (TL). However, in the case of OSL, the insulator/semi-conductor material previously exposed to ionizing radiation is stimulated by light instead of heat^10^,^12^. The electrons trapped in the band gap (due to the previous irradiation) are optically stimulated (usually a blue light), to give ultraviolet emission^10^,^12^. The OSL intensity is collected as a function of time, in a readout window ranging from 270 < λ < 370 nm. It is noteworthy that the OSL emission has energy greater than the excitation energy, due to the energy previously stored by the trapped electron. This contrasts with other luminescence processes, such as photoluminescence^12^.

The OSL signal consists of an exponential decay originating from the release of trapped electrons and the consequent emptying of their respective straps. The OSL intensity can be measured as the initial OSL intensity and/or the integral of the exponential decay curve. Both these parameters are proportional to the ionizing radiation dose previously absorbed by the material. For this reason, OSL is largely used for radiation detection and dosimetry^10^,^12^. Here, all the samples were irradiated with the same dose of radiation (10 Gy), so all the samples should have the same number of trapped electrons. Therefore, the luminescence enhancement can be measured as the ratio between the OSL intensity of the NaCl nanocrystals deposited over the AgNP films and over glass.

The OSL intensity can be described as a sum of *n* exponential decays, as follows:


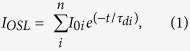


where *I*_0*i*_ is the initial OSL intensity (OSL intensity at t = 0 s) of the *i* trap and is proportional to the initial number of trapped electrons (*n*_0*i*_); and *τ*_*di*_ is a time constant given by:


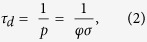


where *p* is the probability that a trapped electron escapes. In turn, *p* is given by the product of the fluency of the incident photon beam (*φ*) and the photoionization cross-section (*σ*) of the trap at the incident wavelength. The probability of electron escape increases with increasing photon fluency (*φ*) and/or the photoionization cross-section (*σ*). For this reason, the decay time (*τ*_*d*_) decreases, and the OSL intensity decays faster. Therefore, analysis of the shape and decay times of the OSL curves provides information regarding the charge de-trapping mechanism and rate.

## Additional Information

**How to cite this article**: Guidelli, E. J. *et al.* Unconventional Increase in Non-Radiative Transitions in Plasmon-Enhanced Luminescence: A Distance-Dependent Coupling. *Sci. Rep.* **6**, 36691; doi: 10.1038/srep36691 (2016).

**Publisher’s note:** Springer Nature remains neutral with regard to jurisdictional claims in published maps and institutional affiliations.

## Figures and Tables

**Figure 1:**
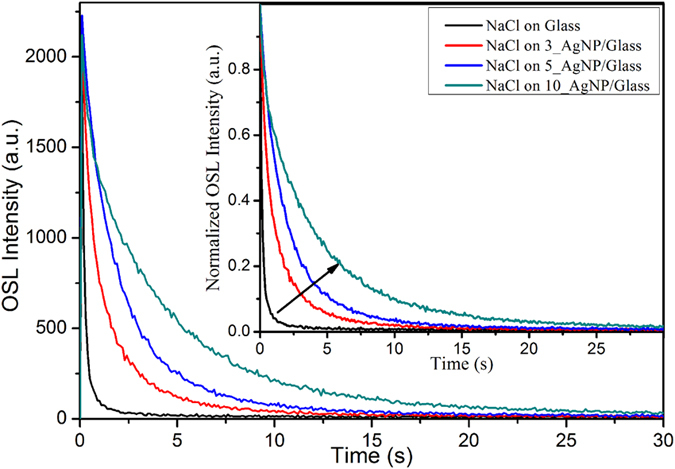
OSL signals for the NaCl nanocrystals deposited over glass and over AgNP films containing 3, 5, and, 10 AgNP/chitosan bilayers. The inset corresponds to the signals normalized by their respective initial intensities.

**Figure 2:**
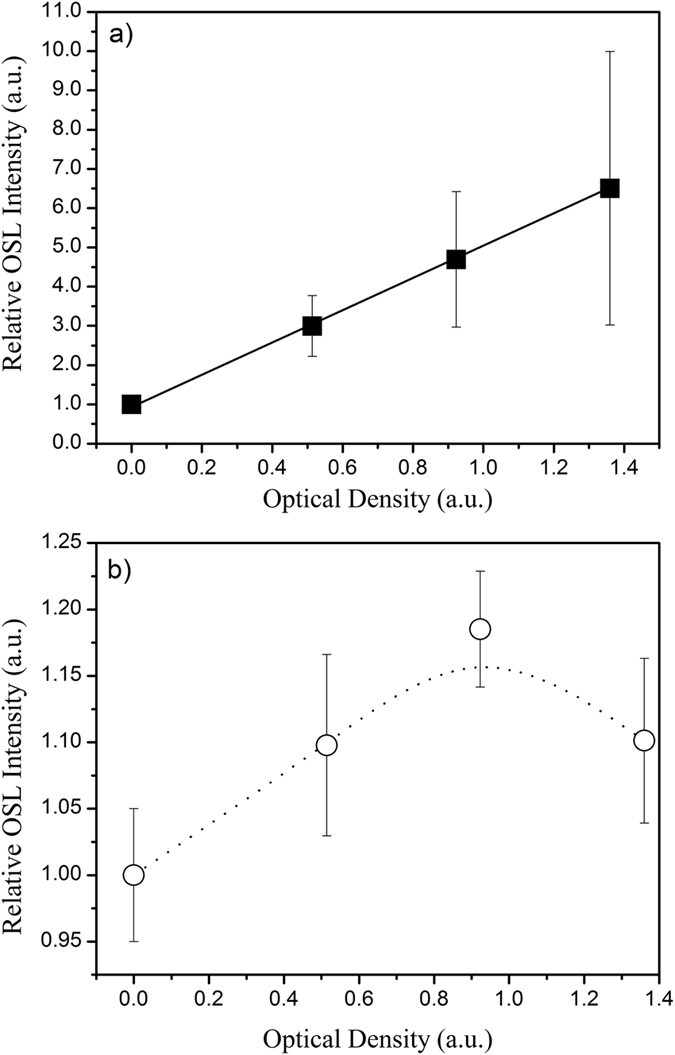
Integral OSL intensity (**a**) and Initial OSL Intensity (**b**) as a function of the optical density of the AgNP films at 470 nm.

**Figure 3:**
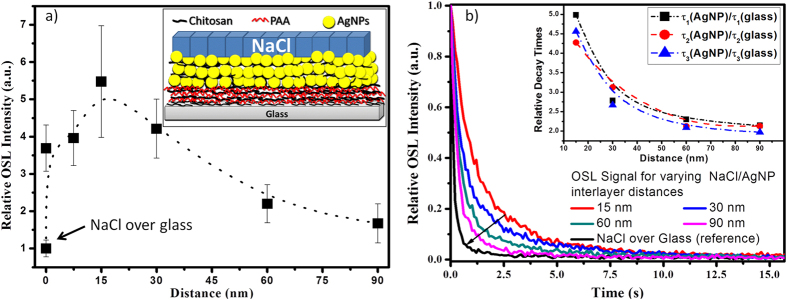
(**a**) OSL intensity (integral) as a function of the distance between the NaCl nanocrystals and the film containing three chitosan/AgNP bilayers. (**b**) OSL signals normalized by their respective initial intensity for the NaCl nanocrystals deposited over glass and over the films containing three chitosan/AgNP bilayers with 1, 2, 4, and 6 chitosan/poly(acrylic acid) interlayers. The inset depicts the three OSL decay times normalized by the values obtained from the NaCl nanocrystals deposited over glass as a function of the distance between the NaCl nanocrystals and the AgNP films.

**Figure 4:**
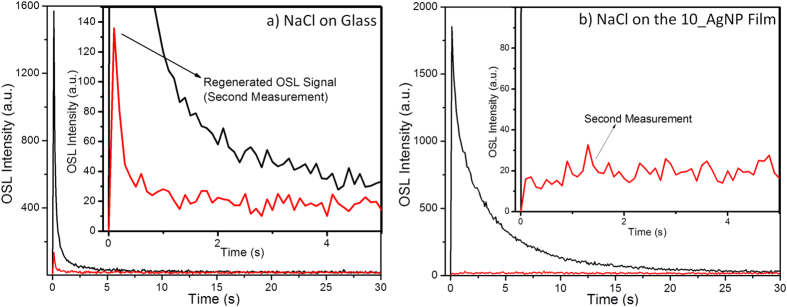
(**a**) First and second measurements of the OSL signal of NaCl nanocrystals deposited over glass. The inset shows the regenerated OSL signal at the second measurement, recorded 10 minutes after a first complete measurement. (**b**) First and second measurements of the OSL signal of NaCl nanocrystals deposited over the film with 10 chitosan/AgNP bilayers. The inset shows that the OSL signal was not regenerated, which suggested that all the electrons/traps were released/emptied under plasmon resonance conditions.
